# Comparative Optimization of Hot Water and Ultrasound-Assisted Extraction of Crude Polysaccharides from Oat (*Avena sativa* L.) for Structural Characterization and Functional Properties

**DOI:** 10.3390/polym18141740

**Published:** 2026-07-16

**Authors:** Nannapat Phosarith, Thanyaporn Siriwoharn, Rattana Muangrat, Suwinai Saengyo, Wachira Jirarattanarangsri

**Affiliations:** 1 Division of Food Science and Technology, Faculty of Agro-Industry, Chiang Mai University, Chiang Mai 50100, Thailand; nannapat_psr@hotmail.com (N.P.); thanyaporn.s@cmu.ac.th (T.S.); suwinai.s@cmu.ac.th (S.S.); 2 Division of Food Engineering, Faculty of Agro-Industry, Chiang Mai University, Chiang Mai 50100, Thailand; rattana.m@cmu.ac.th

**Keywords:** β-glucan, FTIR, response surface methodology, enzyme inhibitor, antioxidant activity, swelling power, monosaccharide composition

## Abstract

This study aimed to evaluate the efficacy of crude polysaccharide extraction from Thai-cultivated oats (*Avena sativa* L.) utilizing hot water extraction (HW) and ultrasound-assisted water extraction (UW) methods. Optimal conditions were determined by a response surface methodology (RSM). The influence of solid-to-liquid ratio, temperature or %amplitude, and extraction time on %yield and beta glucan content was investigated. Under optimal conditions, UW produced a superior %yield (82.72 ± 2.19%) and beta glucan content (1.75 ± 0.87 g/100 g extract) compared to HW (41.32 ± 0.98% and 1.25 ± 0.27 g/100 g extract). This finding may occur from acoustic cavitation, which effectively dismantles the cellular wall structure, supported by FTIR analysis finding more distinct β-glycosidic linkage peaks. SEM analyses indicated a greater surface area dispersion and porosity in UW extract relative to HW extract. Analysis of monosaccharide composition supported the properties of both crude extracts, demonstrating glucose as the predominant component. However, the functional and bioactive characterization demonstrated a distinct trade-off between the two extraction methods. HW extract demonstrated superior swelling capacity (6.2 vs. 2.7 g/g at pH 6.5), enhanced antioxidant activity compared to both ABTS (0.67 ± 0.04 vs. 0.58 ± 0.06 μmol TE/g), DPPH (0.53 ± 0.06 vs. 0.35 ± 0.06 μmol TE/g), and FRAP (0.05 ± 0.02 vs. 0.03 ± 0.01 μmol TE/g), total phenolic content (98.33 ± 7.68 vs. 87.79 ± 3.07 mg GAE/g). The crude extracts from the two methods had selective enzyme inhibitory activity, exhibiting considerable inhibition of α-glucosidase and markedly reduced inhibition of α-amylase. UW demonstrated slightly superior inhibitory activity compared to HW for both enzymes. The findings indicate that the determination of crude polysaccharides should principally take into account the purpose of the final product. UW is preferable for optimizing %yield and beta glucan content. Nevertheless, if the emphasis is on functional attributes like water absorption and antioxidant efficacy, HW has advantages that merit a consideration. This research provides a framework for identifying optimal extraction methods aimed at extracting crude polysaccharides from Thai-cultivated oats for use as a functional ingredient in health food products.

## 1. Introduction

Dietary fiber, regarded as an essential nutrient component following water, protein, fat, carbohydrates, minerals, and vitamins, has garnered significant global interest as a health-promoting ingredient of food [1]. Consequently, the intake of dietary fiber as polysaccharides has garnered significant attention [2]. Oat beta glucan is a prominent polysaccharide, characterized by extensive scientific evidence accumulated over decades, which has been validated by both the Food and Drug Administration (FDA) and the European Food Safety Authority (EFSA) regarding its cholesterol-lowering and glycemic response-regulating properties [3,4]. Beta glucan is a linear polysaccharide consisting of glucose units connected by β-(1 → 3) and β-(1 → 4) glycosidic bonds. This configuration imparts beta glucan its unique functional characteristics. The principal mechanism elucidating its postprandial hypoglycemic action is the augmented gastrointestinal viscosity, which decelerates stomach digestion and diminishes the rate of glucose absorption into the bloodstream [5].

The Thai oat (*Avena sativa* L.: *A. sativa*) strains utilized in this research are cultivated and developed by the Chiang Mai Agricultural Research and Development Center in Thailand. This indicates the possibility of advocating for oats as an alternative economic crop in Thailand’s highlands. However, investigations on the chemical composition and bioactivity of polysaccharides derived from Thai-grown oats are limited in comparison to studies on oat varieties cultivated in the colder climes of Europe and North America, the predominant oat-producing countries globally. Much of the research is concentrated on the extraction and analysis of purified beta glucan from varieties cultivated in temperate regions of Europe and North America, where commercial oat production is predominantly located in Northern Hemisphere nations including Russia, Canada, the United States, Australia, Germany, Finland, and China [6,7,8]. The available literature contains limited data on the qualities of oat polysaccharides cultivated in tropical locations, especially those kinds developed for highland areas in Thailand. The selection of extraction and purification methods profoundly influences the purity, yield, structure, physicochemical qualities, and functional characteristics of beta glucan. However, comparative analyses regarding the impact of various extraction methods, particularly hot water extraction (HW) and ultrasound-assisted extraction (UW), on the characteristics of crude oat polysaccharide are limited. Most of research in this field has focused on alternative raw materials, including the comparison of extraction methods for crude oat polysaccharide from shiitake mushrooms (*Lentinula edodes*), which revealed that UW produced the highest yield of beta glucan through acoustic cavitation, whereas HW preserved structural integrity more effectively [9]. Additionally, a method was developed for extracting beta glucan from oat bran utilizing subcritical water in conjunction with enzymatic digestion [10]. This indicates a distinct information deficiency in comparative analyses of extraction methods for crude oat polysaccharide, particularly from oats cultivated in tropical environments. Investigating the characteristics of polysaccharides from Thai oat cultivars is essential for establishing basic information to facilitate the development of alternative raw materials for the local health food supply. In commercial beta glucan production, HW is the conventional and prevalent method, given that beta glucan exhibits great solubility in hot water. This method commonly requires prolonged extraction durations and substantial thermal energy expenditure [11], which may conflict with the growing trend of environmentally sustainable and energy-efficient extraction practices in the food industry. Over the past decade, UW methods have been extensively utilized in polysaccharide extraction investigation [12] due to the efficacy of acoustic cavitation in disrupting plant cell wall structures, thereby enhancing extraction efficiency and decreasing extraction time relative to conventional methods [13]. Most research on beta glucan primarily focuses on developing methods to achieve high-purity products. This necessitates the elimination of naturally occurring starch and protein impurities using multi-step enzymatic digestion processes, employing α-amylase and glucoamylase in conjunction with protease enzyme removal [11,14,15]. This procedure significantly increases the complexity and expense of large-scale industrial production. However, crude polysaccharide extracts that preserve additional naturally occurring constituents, such as starch and protein, in conjunction with beta glucan may represent a more economically feasible strategy for the development of functional ingredients in food product [16,17] owing to diminished production complexities and costs. The existence of additional components in the crude extract indicates that the functional properties and biological activities found are a result of the interactions among all components in the matrix effect, rather than solely the qualities of beta glucan. This issue is significant and must be addressed and stated clearly when analyzing the experimental data of this sort of crude extract.

This research aims to evaluate the effectiveness of crude polysaccharide extraction methods from Thai *A. sativa* strains using HW and UW methods and applying a response surface methodology (RSM) experimental design to determine the optimal parameters for %yield and beta glucan content. The chemical structure and physical properties of the extracts were analyzed using FTIR, SEM, and monosaccharide composition assessment. Additionally, the functional qualities and biological activities of the crude extracts derived from both procedures were analyzed. The objective is to provide extensive insights into extraction efficiency and its possible applications as functional raw materials in the food industry, while evaluating the viability of industrial-scale production devoid of intricate and expensive purification methods.

## 2. Materials and Methods

### 2.1. Materials and Chemicals

*A. sativa* utilized in this study were procured from the Rice Research Center in Thailand. This particular oat variety was initially imported from the International Maize and Wheat Improvement Center (CIMMYT) in Mexico during the 1980s and has been subsequently evaluated for selective breeding and acclimation by Thai researchers to adapt to local tropical climatic conditions. The oat samples were desiccated at 60 °C prior to grinding using a hammer mill and sieving to get a powder with particle sizes less than 0.5 mm. The samples were defatted by soaking them in 80% ethanol at a solid-to-liquid ratio of 1:50 *w*/*v* and boiling at 90 °C for 2 h. The materials were subsequently desiccated at 60 °C and preserved in vacuum-sealed bags at 4 °C.

Ethanol, methanol, sodium hydroxide, glacial acetic acid, and hydrochloric acid were purchased from RCI Labscan Ltd., Bangkok, Thailand. Folin–Ciocalteu reagent and Potassium bromide (KBr) were purchased from Merck, Darmstadt, Germany. α-amylase (Porcine pancreatic: EC 3.2.1.1), α-glucosidase (Bacillus stearothermophilus: EC 3.2.1.20), and β-Glucan Assay Kit (Yeast and Mushroom) were obtained from Megazyme International (Bray, County Wicklow, Ireland). 2-Chloro-4-nitrophenyl α-D-maltotrioside (CNP-G3) was purchased from Toronto Research Chemicals (TRC, Toronto, ON, Canada). Monobasic sodium phosphate (NaH_2_PO_4_); dibasic sodium phosphate (Na_2_HPO_4_); 1, 1-Diphenyl-2-picrylhydrazyl (DPPH); 2,2′-Azino-bis (3-ethylbenzothiazoline-6-sulfonic acid; TPTZ (2,4,6-tripyridyl-s-triazine); and sodium carbonate (Na_2_CO_3_) were purchased from Sigma–Aldrich (St. Louis, MO, USA). Acarbose and 4-Nitrophenyl-α-D-glucopyranoside were purchased from Macklin, Shanghai, China.

### 2.2. Hot Water Extraction (HW)

The extraction was performed as previously researched with some modifications [18]. 10 mg of A. sativa powder was dissolved with distilled water at varying solid-to-liquid ratios (1:20, 1:30, 1:40 *w*/*v*). The mixture was extracted at varying temperatures (50, 70, and 90 °C) for different durations (15, 30, and 45 min). Following extraction, the samples were centrifuged at 6000 rpm for 10 min at 4 °C to separate the clear liquid from the solid residue. Each aqueous fraction was combined with 95% ethanol (1:2 *v*/*v*) and incubated overnight at 4 °C to facilitate precipitation. The mixture was centrifuged again under the same conditions. The precipitate was gathered, dehydrated in a vacuum oven at 65 °C, and preserved at −18 °C for further analysis.

### 2.3. Ultrasound-Assisted Water Extraction (UW)

UW was performed using an adaptation of a previously researched methodology [19]. 10 g of dried *A. sativa* powder were combined with distilled water at ratios of 1:20, 1:30, and 1:40 (*w*/*v*). The mixture obtained treatment with an ultrasonic device using a 25 mm probe (20 kHz frequency, 750 W peak power, model: SONIC 3, SONIC Corporation, Newtown, CT, USA) at three different amplitudes (50%, 60%, and 70%) for various durations (15, 30, and 45 min) while maintaining a temperature below 60 °C, using an external water bath for temperature control, and conducting the ultrasonic operation in an intermittent mode (9 s on, 1 s off). The sample was centrifuged at 6000 rpm for 10 min at 4 °C. The transparent liquid fraction was combined with 95% ethanol in a 1:2 volume ratio and incubated overnight at 4 °C. Centrifugation was performed under the same conditions. The crude extract precipitate was dried at 65 °C in a vacuum oven and stored at −18 °C.

### 2.4. Experimental Design

The optimization of crude polysaccharide from A. sativa was performed using Response Surface Methodology (RSM). A three-factor, three-level Box–Behnken design (BBD) was employed to evaluate the influence of critical extraction parameters on beta glucan content. The HW extraction method utilized independent variables including the solid-to-liquid ratio (1:20–1:40 *w*/*v*), extraction temperature (50–90 °C), and extraction duration (15–45 min). In the UW extraction method, the temperature factor was displaced by %amplitude (50–70%) to more accurately represent ultrasonic conditions. All assessments, including the central point, were conducted three times. The %yield and beta glucan content (g/100 g) provided the response variable (according to Table 1 and Table 2).

Statistical analysis was performed using Design Expert 10 software (trial version; Stat-Ease Inc., Minneapolis, MN, USA). Experimental data were fitted to a quadratic model corresponding to the response. The standard representation of the quadratic equation is shown in Equation (1):

*Y* = *b*_0_ + ∑^(k)i=1^ *b*_i_*x*_i_ + ∑^(k)i=1^ ∑^(k)^_j_^=i^ *b*_ij_*x*_i_*x*_j_ + ∑^(k)i=1^ *b*_ii_*x*_i_^2^ (1)

where Y is the dependent variable; b_0_ is a constant; and b_i_, b_ii_, and b_ij_ are the coefficients estimated by the model; x_i_ and x_j_ are the degrees of the independent variable.

### 2.5. Measurement of Beta Glucan Content

Quantification was performed using a Megazyme mixed beta glucan assay kit (Megazyme, Bray, Ireland) according to the manufacturer’s guidelines, with minor modifications. Samples from 80 to 120 mg were added to 200 µL of water (50% *v*/*v*) ethanol, then followed by 4 mL of 20 mM sodium phosphate buffer solution (pH 6.5), following an incubation in a boiling bath. All other steps were outlined in the user manual presented in the set of materials.

### 2.6. Fourier-Transform Infrared (FT-IR) Spectroscopy

The FTIR spectrum was recorded using a JASCO FT/IR 4700 spectrometer (JASCO Inc., Easton, MD, USA) operating in KBr mode. All data were collected in the range of 400 to 4000 cm^−1^.

### 2.7. Scanning Electron Microscopy (SEM) Analysis

The samples were coated with a thin layer of gold under low pressure and examined using a scanning electron microscope (SEM, JSM-IT300, JEOL Ltd., Tokyo, Japan) at an accelerating voltage of 3 kV and a magnification of 1500×.

### 2.8. Determination of Monosaccharide Composition

The method of analysis was modified from previous studies [20]. The sample was meticulously pulverized and weighed 1.42 g. An 8 mL solution of sulfuric acid at a concentration of 3.89% (*w*/*v*) was then added for the hydrolysis of polysaccharides. The sample performed acid hydrolysis in an autoclave at 95 °C for 1 h. Following hydrolysis, the mixture was transferred to a cylinder and diluted to a volume of 14 mL using acetonitrile. The solution was cooled to −20 °C for 1 h to facilitate precipitation. The solution was centrifuged at 10,000 rpm for 2 min and filtered through a nylon membrane filter before analysis. The examination of sugar content and concentration was conducted utilizing an Agilent 1260 Infinity II HPLC system (Agilent Technologies, Santa Clara, CA, USA) equipped with a Shodex Asahipak NH_2_P-50 4E column (4.6 × 250 mm, Showa Denko, Tokyo, Japan). The sample injection volume was 6.0 μL, the column temperature was maintained at 30 °C, and the mobile phase consisted of acetonitrile and water in a 70:30 (*v*/*v*) ratio. The flow rate was 1.0 mL/min, the analysis duration was 15 min, and a Refractive Index (RI) detector was employed. The identification of sugar types was conducted by comparing retention periods with standard compounds under identical analytical circumstances. The retention times for each sugar were as follows: arabinose at 5.3–5.4 min, xylose at 5.5–5.6 min, mannose at 6.4–6.5 min, galactose at 6.6–6.7 min, and glucose at 6.9–7.0 min. The sugar type in the sample was verified by comparing the retention durations of the observed peaks with reference substances, and quantification was conducted using external standard calibration. All measurements were performed in triplicate to ensure analytical precision.

### 2.9. Swelling Power Determination

0.1 g of sample was dissolved in 10 mL of phosphate buffer solution at various pH levels (3, 4, and 6.5) and incubated for 2 h at 37 °C. The sample was centrifuged at 10,000 rpm for 5 min, after which the residual material was collected and weighed. Swelling power refers to the increase in weight [9], determined by the formula shown in Equation (2).

Swelling power = (W2 − W1)/W1 × 100(2)

### 2.10. Antioxidant Activity Assays

#### 2.10.1. ABTS Radical Scavenging Activity

The ABTS scavenging activity was slightly modified from previous studies [21]. A standard solution of ABTS+ was prepared by combining 7 mM ABTS solution with 2.45 mM persulfate solution in equal proportions and incubating in the dark at ambient temperature for 16 h. The ABTS+ stock solution was diluted with distilled water to achieve an absorbance of 0.7 ± 0.02 at 734 nm. Next, 50 µL (0.1 g/10 mL) of the sample extract was combined with 100 µL of ABTS solution. Absorbance values were measured after 8 min at 734 nm using a 96-well microplate reader (Tecan Group Ltd., Männedorf, Switzerland). The scavenging activity was measured as a percentage based on Equation (3):

%Scavenging activity = (Acontrol − Asample)/Acontrol × 100 (3)

#### 2.10.2. DPPH Radical Scavenging Activity

The free radical scavenging activity of crude polysaccharide was conducted with minor modifications to previous research [22]. A 0.1 mM DPPH free radical solution in 70% methanol was produced, and 160 µL of this solution was combined with 40 µL of an aqueous sample solution (0.1 g/10 mL). The solution’s absorbance was evaluated at 517 nm utilizing a 96-well microplate reader. Methanol served as the control, whereas Trolox functioned as the standard. The DPPH scavenging capacity was determined using the subsequent Equation (3).

#### 2.10.3. Ferric-Reducing Antioxidant Power (FRAP) Assay

The total antioxidant activity of polysaccharides was assessed using the FRAP assay, which evaluates the ferric reducing capacity of crude polysaccharides [23]. The FRAP solution was prepared by combining 10 mL of 300 mM acetate buffer at pH 3.6 with 10 mM TPTZ in 1 mL of 40 mM HCl and 1 mL of 20 mM FeCl_3_·6H_2_O. The FRAP solution was elevated to 37 °C. Then combined 150 µL of the freshly prepared FRAP solution with 50 µL (0.1 g/10 mL) of the sample extract. The solution was incubated at 37 °C for 10 min in a water bath. Absorbance was quantified at 593 nm in comparison to the blank solution after 4 min. The FRAP value was determined based on the micromoles of Fe^2+^ equivalents per 100 g of material. Utilizing the Fe^2+^ calibration graph.

### 2.11. Total Phenolic Compound (TPC)

The total phenolic content (TPC) in crude polysaccharide was evaluated following prior study with certain adjustments [24]. 25 µL of each extract (0.1 g diluted in 10 mL) was combined with 125 µL of 0.2 M Folin–Ciocalteu reagent. Following a 10 min incubation, 125 µL of 7.5% Na_2_CO_3_ was introduced into the mixture and incubated for an additional 30 min. Absorbance was quantified at 750 nm utilizing a 96-well microplate reader. The total phenolic content (TPC) was quantified as gallic acid equivalent (GAE).

### 2.12. α-Amylase Inhibition Activity

The inhibitory action of alpha-amylase was evaluated according to a published method with slight modifications [25]. 50 μL of extract (containing 0.25 to 4.0 mg/mL) were combined into the mixture with 100 μL of CNP-G3 and incubated at 37 °C for 10 min. Next, 50 μL of alpha-amylase solution (0.5 U/mL, 20 mM sodium phosphate buffer with 6.7 mM NaCl, pH 6.9) was added and incubated at 37 °C for 15 min. The absorbance of the reaction solution was subsequently quantified at 405 nm utilizing a microplate reader. Acarbose served as a positive control. The inhibitory activity of alpha-glucosidase was determined as follows:

%Inhibition = (Acontrol − Asample)/Acontrol × 100 (4)

### 2.13. α-Glucosidase Inhibition Activity

The inhibiting impact of the materials on α-glucosidase was measured using a minor modification [9]. 50 μL of the sample (containing 0.5 to 8.0 mg/mL) were combined with 50 μL of α-glucosidase solution (1 U/mL in 0.1 M phosphate buffer, pH 6.8) and incubated at 37 °C for 15 min. Next, 50 μL of pNPG solution (4 mM in 0.1 M phosphate buffer, pH 6.8) was included into the mixture and incubated at 37 °C for 30 min. The absorbance of the reaction solution was quantified at 405 nm utilizing a microplate reader. Acarbose served as a positive control. The inhibitory activity of α-glucosidase was determined according to Equation (4).

### 2.14. Statistical Analysis

The statistical analyses of the results were conducted by SPSS software (version 23.0, SPSS Inc., Chicago, IL, USA) and were presented as mean values with standard deviations. Duncan multiple comparison tests were used (*p* < 0.05) to evaluate the differences between the means.

## 3. Results and Discussion

### 3.1. Extraction Optimization by RSM

The results of crude polysaccharide extraction from *A. sativa* using the HW and UW procedures across 15 experimental conditions indicated that the UW methods produced a significantly greater average %yield compared to the HW method. The UW methods produced a yield ranging from 59.72% to 83.66%, whereas the HW method yielded between 23.85% and 49.03%, as illustrated in Table 3. Additionally, the beta glucan content in crude polysaccharide obtained by the UW methods equaled that of the HW method. The UW methods produced BG content between 1.32 and 1.75 g/100 g extract, whereas the HWE method resulted in 0.84 to 1.27 g/100 g extract, indicating an average enhancement of almost 59%.

The experimental findings align with the mechanism involving high-frequency sound waves that may be ascribed to generating acoustic cavitation in the solvent medium. The collapse of cavitation produces microjets and mechanical shear stresses that disrupt the cell wall structure of *A. sativa* and induce further relaxing of the cell matrix [9,23,26]. In contrast to heating alone in the HW method, this mechanical force could be linked to enhances the surface area contact between the extraction substrate and the plant tissue, allowing the more efficient and rapid diffusion of polysaccharides confined inside the cell wall and endosperm structures into the medium [27]. This indicates that the UW method produces a markedly superior %yield compared to the HW method. The elevated beta glucan content in the UW extract can be attributed to the same mechanism; specifically, beta glucan in oats predominantly resides in the endosperm cell wall, where it is coupled to other structural components. Cavitation induced by high-frequency sound waves disintegrates these bonds more efficiently than traditional heating methods. This might be attributed to an increased release of beta glucan into the extract [28].

### 3.2. Analysis of Variance (ANOVA) and the Quality of Statistical Models

Response surface methodology (RSM) analysis indicated notable disparities in extraction efficiency between HW and UW methods for crude polysaccharide and beta glucan derived from *A. sativa*. The investigation indicated that each model was appropriate for examining factors influencing yield and identifying optimal conditions. the response variable and the test variables were associated with the following second-order polynomial Equations (5)–(8):

HW: Y = 38.63 + 1.78A + 5.70B + 5.90C + 0.59AB + 1.43AC − 1.38BC − 0.87A^2^ + 2.69B^2^ − 5.37C^2^ (5)

UW: Y = 73.43 − 0.67A + 4.53B + 3.88C − 1.068AB − 1.76AC − 0.34BC + 0.37A^2^ − 5.54B^2^ + 2.00C^2^ (6)

HW: BG = 0.87 − 0.62A + 0.08B + 0.04C − 0.003AB − 0.006AC − 0.04BC + 0.08A^2^ + 0.17B^2^ − 0.016C^2^ (7)

UW: BG = 1.65 − 0.02A + 0.08B + 0.16C + 0.01AB − 0.004AC +0.002BC − 0.01A^2^ + 0.02B^2^ − 0.11C^2^ (8)

ANOVA analysis of quadratic polynomial models for the responses, particularly %Yield and beta glucan content derived from HW and UW, indicated that all models were statistically significant (model *p* < 0.05), with *p*-values of 0.0208, 0.0393, 0.0467, and 0.0021 for Yield-HW, BG-HW, Yield-UW, and BG-UW, respectively. This confirms that the developed models can substantially elucidate the correlation between the examined variables and the replies. Moreover, the lack of fit values for all four models were not statistically significant, suggesting that quadratic polynomial models effectively characterize the relationship between independent variables and responses within the examined experimental range without substantial model deviation, as illustrated in Table 4 and Table 5.

All models had elevated coefficients of determination (R^2^) ranging from 0.8989 to 0.9731, signifying a notable capacity to elucidate data variance. The BG-UW model exhibited the greatest R^2^ (0.9731) and adjusted R^2^ (0.9247) within the groups, along with the lowest coefficient of variation (C.V.) (2.53%), indicating a significant degree of precision and reliability. In contrast, the Yield-UW model exhibited the lowest Adjusted R^2^ (0.7170) among the groups, despite its low C.V. of 4.4%. In the HWE method, the linear variables that significantly influenced %yield were temperature and extraction time, whereas the solid-to-liquid ratio exhibited no significant impact. Moreover, the squared term of extraction time was significant, suggesting a nonlinear link between extraction time and %yield. The %yield may initially rise over time but will then peak and either decline or stabilize once the extraction duration is beyond the ideal threshold. This may result from the breakdown of certain extracted polysaccharides due to prolonged exposure to high temperatures [6,9]. In contrast, for the UW method, the variables that significantly influenced %yield were %amplitude and extraction time, similar to the HW method. The extraction ratio did not significantly impact either approach, suggesting that within the examined ratio range, the volume of extraction medium utilized is adequate for dissolving and transporting polysaccharides from plant tissue and hence is not a limiting factor in the process. The square term of %amplitude was significant in the Yield-UW model, indicating a comparable nonlinear relationship. Increasing the amplitude of sound waves to extremely high levels may not proportionately enhance %yield, potentially due to saturation effects or the creation of very thick air bubbles that obstruct the transfer of sound wave energy into the medium [29]. Considering parameters influencing beta glucan content, the HW method indicated that both the ratio and temperature considerably impacted beta glucan content, although extraction time did not exert a significant effect; in contrast, time had a pronounced influence on %yield. This suggests that the determinants of %yield and beta glucan content may differ. The squared term of temperature was significant (*p* < 0.05), indicating a nonlinear relationship between temperature and beta glucan content, which aligns with the heat-sensitive characteristics of polysaccharides. Exceeding optimum temperatures may result in degradation of the beta glucan structure or the initiation of side reactions [30]. In the UW approach, both %amplitude and extraction duration significantly influence the quantity of beta glucan. Time is the most significant factor affecting the release of beta glucan during ultrasonic extraction. Additionally, the squared term of the extraction time is relevant, indicating a distinct curvilinear relationship. This phenomenon is elucidated by the observation that prolonging the initial cavitation duration significantly enhances the releasing of beta glucan from the cellular matrix. However, when the duration exceeds the optimum threshold, the mechanical shear stress and heat accumulation resulting from continuous ultrasonic waves may induce depolymerization of polysaccharide chains [31].

### 3.3. Optimization Analysis of Extraction Parameters Affecting Beta Glucan Content

**Figure 1:**
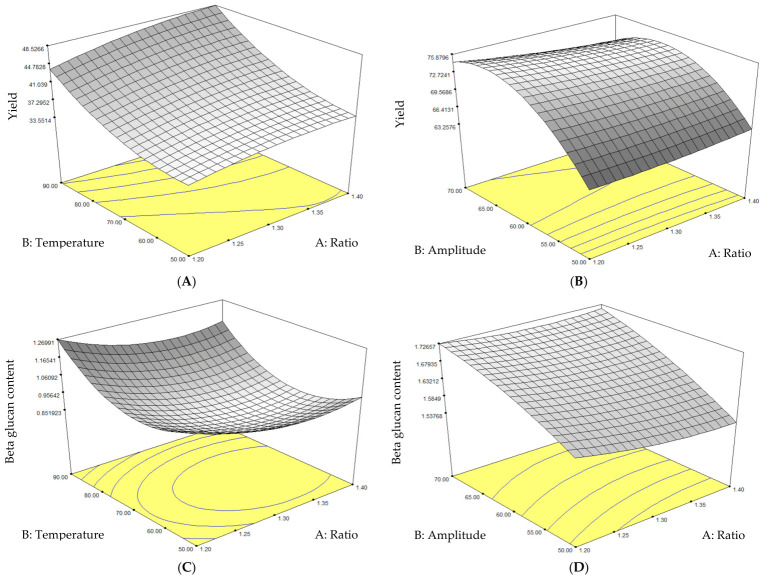
Response surface plots showing the effects of extraction parameters on yield extraction and beta glucan content from *A. sativa* under: (**A**) yield: hot water extraction, (**B**) yield: ultrasound-assisted water (**C**) BG: hot water extraction, and (**D**) BG: ultrasound-assisted water.

Figure 1A demonstrates the influence of temperature and ratio on %yield in the HW method, indicating that the response surface is a markedly curved plane. %Yield consistently rises with escalating temperature from 50 to 90 °C, but variations in ratio exhibit minimal influence on the surface slope. The ANOVA study results indicate that temperature was statistically significant (*p* < 0.05), while ratio was non-significant, indicating that thermal energy is the principal factor influencing HW mass transfer. Elevated temperatures diminish the viscosity of the medium and augment the kinetic energy of water molecules, hence enhancing the efficiency of polysaccharide diffusion from plant tissue to the medium [32], as illustrated in Figure 1.

In contrast, Figure 1B, showing the %amplitude and its relation to the %yield effect in the UW method, presents a curved surface. The maximum %yield appears at elevated ratios with little %amplitude and generally declines as %amplitude rises within specific ratio ranges. This behavior can be elucidated by the phenomenon of cavitation saturation; specifically, at amplitudes surpassing the optimal threshold, air bubbles produced by acoustic cavitation may assemble strongly, potentially forming acoustic shielding that could obstruct the effective transmission of sound wave energy into the liquid medium [33]. Consequently, the %yield does not appear to exhibit a linear increase with rising amplitude. Figure 1C illustrates the HW response surface for beta glucan content, characterized by a concave bowl shape, with the minimum beta glucan content located at the midpoint of the investigated temperature and ratio ranges. In the low to moderate temperature range, elevating the temperature enhances the diffusion and dissolution of beta glucan from the cell wall structure. However, when the temperature surpasses the optimum threshold, thermal degradation or ancillary processes may transpire, diminishing the solubility of beta glucan [34]. Furthermore, image 1D demonstrates the impact of %amplitude and the ratio to beta glucan content in the UW method, presenting a smooth inclined plane surface, where beta glucan content rises consistently with increasing %amplitude. The disparity in surface patterns between images 1C and 1D is relevant, suggesting that the mechanism of cellular structure degradation by using mechanical shear from ultrasonic cavitation in the UW method may exhibit reduced sensitivity to thermal variations compared to direct contact heating in the HW method. This might lead to diminished beta glucan degradation within the examined amplitude range relative to the temperature scenario in the HW method, which could potentially be attributed to the brief duration of the cavitation process. This short duration may result in less heat accumulation and dissipation in the system compared to prolonged heating [35]. The higher yield observed in UW may be due to increased mass transfer; however, differences in temperature and extraction time may also contribute to this effect.

### 3.4. Verification of the Predicted Optimal Extraction Conditions

Model validation was conducted at the predicted optimum conditions for both extraction methods, as demonstrated in Table 6. The experimental results demonstrated remarkable concordance between the predicted and actual values. In the HW method, under optimal conditions, the model predicted a yield of 44.23%, while the actual yield was 41.32 ± 0.98%, resulting in an error of merely 6.58%. The elevated accuracy signifies the model’s dependability in predicting ideal operating circumstances. In the UW method, under optimal conditions, the model forecasted a yield of 83.36%, while the actual yield was 82.72 ± 2.19%, indicating a minimal error of 0.77%. The superior accuracy of the UW model may result from enhanced process parameter regulation and reduced process variability. Both methods for beta glucan measurement demonstrated remarkable concordance between anticipated and actual levels, with errors of 1.57% and 0.57%, respectively.

### 3.5. FTIR Analysis

**Figure 2:**
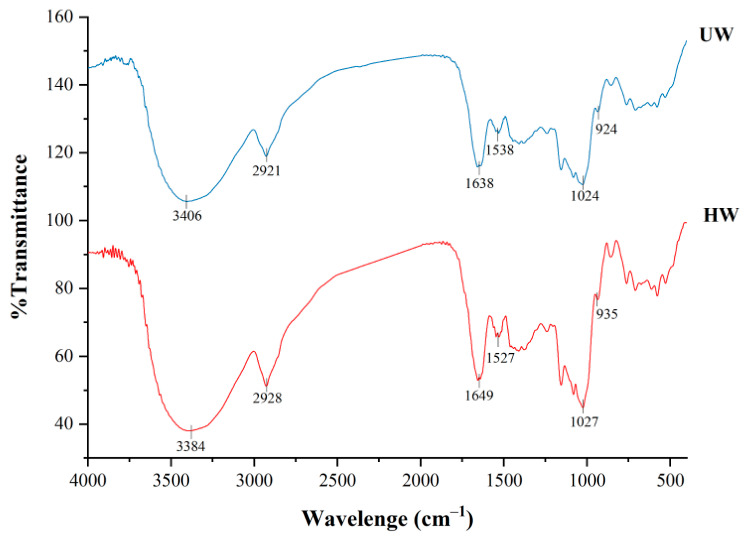
FTIR spectrum of isolated crude polysaccharides from different conditions.

FTIR analysis demonstrated that various extraction methods may have influenced the functional group structure of the crude polysaccharide in different structures, despite the overall peak patterns of both methods being analogous, suggesting similar fundamental chemical compositions of the extracts as illustrated in Figure 2.

The primary distinction between the two methods appears at the O-H stretching peak, where UW exhibits a peak at 3406 cm^−1^ and HW at 3384 cm^−1^. The approximate 22 cm^−1^ blue shift may result from mechanical shear and acoustic cavitation in the UW process, which disrupts intermolecular hydrogen bonds, perhaps resulting in a less ordered hydrogen bond network and elevated peak frequencies. In contrast, the uniform heating procedure in the HW approach may facilitate the preservation of a more comprehensive and organized hydrogen bond network [36,37]. After examining the amide I (HW: 1649 cm^−1^; UW: 1638 cm^−1^) and amide II (HW: 1527 cm^−1^; UW: 1538 cm^−1^) regions, it is obvious that the amide I peak in the UW range is slightly displaced towards a lower frequency compared to the HW range. This may indicate partial protein denaturation, wherein mechanical energy from cavitation may induce a breakdown of the protein structure, resulting in the C=O groups of the amide vibrating at a slightly lower frequency [38,39]. Furthermore, in the fingerprint area, peaks at 1024–1027 cm^−1^ and 924–935 cm^−1^ indicate that C-O and C-O-C glycosidic bonds and β-glycosidic linkages, respectively, exhibit greater absorption strength in the UW spectrum than in the HW spectrum. The elevated intensity of these peaks in the UW extract serves as spectroscopic evidence indicating a greater proportion of polysaccharides with β-glycosidic bonds [40], further supporting the previously reported finding that the UW extract (1.75 ± 0.87 g/100 g extract) contains a higher content of beta glucan than the HW extract (1.25 ± 0.27 g/100 g extract). while comparing the FTIR spectra of the extracted polysaccharides with previous studies of oats cultivated in cold environments, the principal peaks indicating glycosidic C–O–C stretching were observed within a comparable range, specifically around 1024–1027 cm^−1^ in Thai oat samples, in contrast to 1032 cm^−1^ reported in Russian oats. This consistency may indicate that the beta glucan backbone in oats remains rather stable across different growing environments [41,42].

### 3.6. SEM Analysis

**Figure 3:**
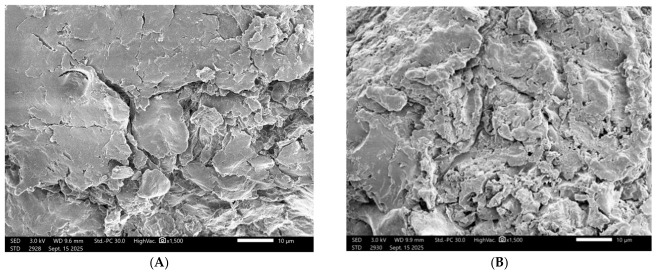
Scanning Electron Microscopy (SEM) images of crude polysaccharides extracted using different extraction methods: (**A**) HW, and (**B**) UW. All images were taken at 1500× magnification, scale bar = 10 µm.

Figure 3 illustrates potential differences between HW and UW, aiding in the examination of the equilibrium characteristics of the crude polysaccharide. The identified structural component appears to correlate with the quantitative analysis detailed in the previous section. The HW extract (Figure 3A) exhibits a flat, plate-like matrix with irregular fractures, and the predominant structure appears to extend vertically [43].

These characteristics may arise from the consistent heating procedure in the HW method, which partially degrades the cell wall while maintaining the integrity of the polysaccharide matrix structure. The FTIR data suggest that the O-H stretching at 3384 cm^−1^ of the HW extract exhibits greater order compared to the UW extract, which also corresponds to an increased swelling power. A more continuous and denser matrix structure may correlate with an enhanced capacity to hold water molecules inside it [44]. In contrast, the UW extract (Figure 3B) has a distinctly broken surface characterized by numerous fissures and cavities dispersed throughout. This feature serves as structural evidence potentially linked to an acoustic cavitation mechanism, which produces greater mechanical shear and micro-shock waves compared to the HW heating method [45]. This could potentially be ascribed to an augmented contact surface area between the plant tissue and the extraction medium, which might subsequently contribute to the observed increase in % yield within the UW approach compared to the HW method (82.72 ± 2.19% vs. 41.32 ± 0.98%) and an increased beta-glucan content (1.75 ± 0.87 vs. 1.25 ± 0.27 g/100 g extract), as previously studied. Moreover, the excessive shear stress may significantly compromise the integrity of the cell wall structure [46]. Additionally, it may induce chain depolymerization, potentially linked to the markedly decreased swelling capacity of the UW extract compared to HW. Smaller chains exhibit diminished capacity to establish a three-dimensional network for water retention [6], corroborated by FTIR results indicating a blue shift of the O-H stretching peak in UW (3406 vs. 3384 cm^−1^), potentially signifying a decreased order of the hydrogen bond network [47].

### 3.7. Monosaccharide Composition Analysis

**Figure 4:**
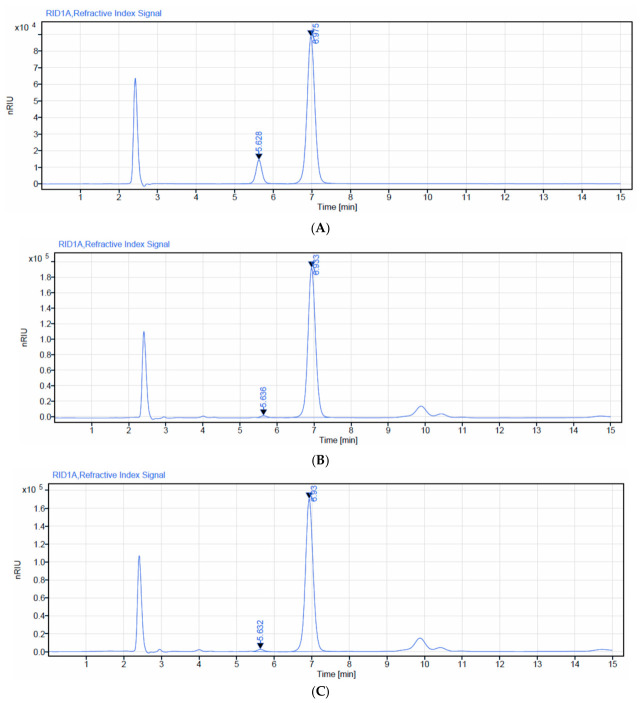
HPLC chromatograms of monosaccharide standards and crude oat polysaccharide samples: (**A**) mixed standards consisting of xylose (6 g/L) and glucose (24 g/L), (**B**) hot water extraction (HW) sample and (**C**) ultrasound-assisted water extraction (UW) sample.

The analysis of monosaccharide content in crude polysaccharides derived from *A. sativa* using HPLC analytical methods is presented in Figure 4A,B. The calibration curves of xylose and glucose exhibited excellent linearity, with correlation coefficients (R^2^) of 0.9999 and 0.9998, respectively. Triplicate analysis (n = 3) showed low standard deviations, indicating good repeatability and precision of the method. Both HW and UW extracts exhibited markedly elevated levels of glucose as the primary component. HW extract had 597.56 ± 1.21 mg/g of glucose and 11.88 ± 0.10 mg/g of xylose, while UW extract contained 524.89 ± 1.73 mg/g of glucose and 13.72 ± 0.20 mg/g of xylose.

The high content of glucose relative to other monosaccharides in this crude extract might be attributed to the characteristics of the high-temperature water extraction method. Cereal polysaccharides could potentially induce starch gelatinization inside *A. sativa* tissue, which may lead to the extraction of gelatinized starch alongside beta glucan as a crude extract. This co-extraction phenomenon has been suggested in several previous studies [48]. High-temperature extraction methods, such as water extraction, alkaline extraction, and subcritical extraction, have been associated with starch gelatinization and potential contamination with starch and beta glucan, which could subsequently diminish the purity of the final product [6,49]. Since glucose is the basic monosaccharide of both starch (α-1,4 and α-1,6 glycosidic linkages) and beta glucan (β-1,3 and β-1,4 glycosidic linkages) [50], the elevated glucose levels seen in this experiment should not be construed as the complete beta glucan content, but instead represent the aggregate of polysaccharides that include glucose [51]. This aligns with previously reported results concerning beta glucan content, which revealed that actual beta glucan content in the crude extract was merely 1.00–1.59 g/100 g extract, considerably lower than the total glucose proportion identified by HPLC. This verifies that most of the glucose in the sample derives from the contaminated starch, rather than exclusively from beta glucan. Regarding xylose, detected in relatively low concentrations in both samples (11.88–12.96 mg/g), this finding might be attributed to xylose being the primary constituent of arabinoxylan, a hemicellulose present in the cell wall of oats in significantly lesser quantities than beta glucan and starch [52]. A comparison of the two extraction methods revealed that the HW method produced slightly elevated glucose content compared to the UW method, while exhibiting a reduced xylose content. These findings may indicate variances in the extraction mechanisms; specifically, extended continuous heating in the HW method may enhance gelatinization and starch dissolution more significantly than the UW method, which primarily employs mechanical shear from cavitation to disrupt cell structures, while the influence of heat on starch is comparatively minimal [53,54]. The experimental results validate that this research focused on investigating crude polysaccharide as a functional ingredient, preserving natural beta glucan, starch, and protein, as proposed in the research objective, rather than examining purified beta glucan, which would necessitate further enzymatic starch removal. Furthermore, the variation in monosaccharide composition between HW and UW samples may not solely derive from the extraction method, but also from variations in processing parameters, including temperature and extraction time. However, comprehensive method validation, including LOD, LOQ, and recovery, was not conducted and should be addressed in future studies.

### 3.8. Swelling Power Analysis

**Figure 5:**
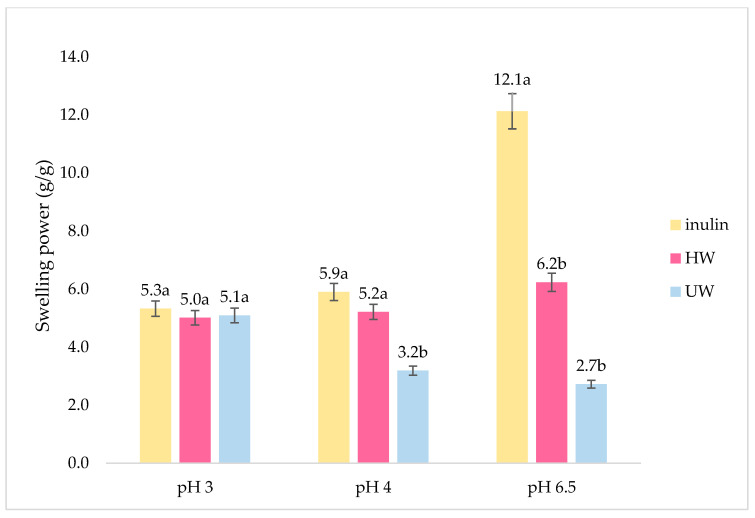
Swelling power of crude polysaccharides extracted from *A. sativa* using different extraction methods. Different letters (a, b) above the bars indicate significant differences (*p* < 0.05) according to Duncan’s multiple range test.

The evaluation of the swelling power of crude polysaccharide from *A. sativa*, extracted using HW and UW methods, in comparison to inulin as a reference standard at pH levels of 3, 4, and 6.5, indicated a propensity for swelling power in conjunction with elevated pH across all samples. Inulin exhibited an increase in swelling power from 5.3 g/g at pH 3 to 12.1 g/g at pH 6.5. The HW and UW samples exhibited a notable disparity, with HW rising marginally from 5.0 to 6.2 g/g and UW declining from 5.1 to 2.7 g/g when pH escalated from 3 to 6.5, as illustrated in Figure 5.

At pH 3, a markedly acidic environment, the swelling power of the three samples did not vary considerably, suggesting that under such conditions, the polysaccharide structure of the samples demonstrated similar water absorption characteristics. This may result from the equal strength of hydrogen bonding among polysaccharide chains under acidic circumstances [55], hence constraining the swelling capacity to a uniform level across all samples. As the pH increased to 4 and 6.5, the disparities across the samples were more pronounced. At pH 4, the UW was markedly inferior to HW and inulin (*p* < 0.05). The disparity was most evident at pH 6.5, with UW measuring a mere 2.7 g/g, in contrast to HW at 6.2 g/g and inulin at a maximum of 12.1 g/g. The experimental results indicate that samples extracted using the HW method consistently demonstrated superior swelling power and enhanced stability compared to those extracted using the UW method across all pH conditions, aligning with research that compares conventional, chemical, and ultrasound methods for crude polysaccharide extraction from *Lentinula edodes*. This comparison showed a similar trend; however, the UW method achieved the highest beta glucan content due to the effectiveness of acoustic cavitation in releasing polysaccharides from the tissue, while the HW method maintained the superior structural integrity of the polysaccharide, resulting in improved and more consistent swelling power across all evaluated pH conditions. This result signifies enhanced water retention capability [9]. The mechanisms defining this phenomenon are associated with the previously described SEM and FTIR analysis findings. The mechanical shear forces and localized shock waves produced by acoustic cavitation in the UW process, while effective in disrupting cell walls and enhancing beta glucan extraction, may also result in polysaccharide chain depolymerization and a decrease in the molecular weight of beta glucan [56]. The swelling and water-holding properties of polysaccharides are directly correlated with molecular weight and the three-dimensional network structure that entraps water molecules within structural voids. Consequently, a reduction in polysaccharide chain length due to fracture may diminish the capacity to form an effective water-retaining network [57]. This phenomenon leads to a reduced swelling power of UW samples relative to HW samples, particularly at elevated pH levels, where the polysaccharide structure inherently loosens, thereby amplifying the effects of chain fracture from the UW process compared to low pH, where the structure remains tightly bound by strong hydrogen bonds. The propensity for swelling power to increase with elevated pH across all samples could potentially be attributed to the possibility that hydrogen bonds maintaining the polysaccharide structure in acidic circumstances appear to be relatively more robust than those in neutral or mildly alkaline environments. As the pH nears neutrality, hydrogen bonds and interchain interactions weaken, rendering the polysaccharide structure more pliable and enabling it to absorb additional water molecules [58]. This behavior aligns with the characteristics of most water-soluble polysaccharides. Furthermore, environmental variables may further affect the structural features of beta glucan. Previous research indicates that higher precipitation correlates with a reduced molecular weight of beta glucan owing to enhanced enzymatic breakdown [59]. Considering that Thai-grown oats are farmed in tropical conditions characterized by heavy precipitation, the beta glucan isolated in this study may intrinsically exhibit a lower molecular weight compared to oats planted in temperate or cold regions. Consequently, the relatively reduced swelling capacity noted, especially in UW samples, may stem from a synergistic influence of environmental factors and extraction-induced degradation. In contrast, oats grown in cooler regions with reduced precipitation tend to preserve higher molecular weight β-glucan, resulting in enhanced functional qualities including swelling power [60]. In addition to these structural considerations, the studied functional attributes of this crude extract arise from the synergistic effects of beta-glucan, phenolic compounds, and other combined extracted compounds, rather than from beta glucan alone. This finding represents a constraint of this study and will be remedied through future purifying projects.

### 3.9. Antioxidant Activity

The analysis of antioxidant activity using ABTS, DPPH, and FRAP methodology, alongside the assessment of total phenolic content (TPC), revealed consistent patterns across all indicators. HW samples consistently exhibited superior values compared to UW samples across all parameters, as shown in Table 7.

This experiment reveals a pattern that contradicts the %Yield and beta glucan content comparisons presented in the preceding section, which indicated that the UW approach produced much higher %yield and beta glucan content than the HW method. Although the UW method demonstrated superior efficiency regarding yield and beta glucan content, it had markedly reduced antioxidant activity and phenolic component content compared to the HW method. This apparently paradoxical phenomenon could potentially be explained by the hypothesis that the antioxidant efficacy of crude polysaccharide extract may not solely rely on the beta glucan content, but could also be influenced by the quantity and state of phenolic chemicals co-extracted within the crude extract. This aligns with research comparing crude polysaccharide extraction methods derived from *Lentinula edodes* using hot water, hot alkaline, ultrasound-assisted water, and ultrasound-assisted alkaline, which revealed a comparable pattern. Although the UW method produced the highest beta glucan content across the groups, it had the lowest antioxidant activity. This suggests that the antioxidant activity of the extract is not exclusively reliant on the quantity of beta glucan [9,61]. The underlying mechanism behind this phenomenon may be associated with the characteristics of phenolic compounds, which appear to be affected by mechanical shear and localized shock waves produced by acoustic cavitation. While cavitation is widely considered effective at disrupting cell wall structures and enhancing polysaccharide release from oat tissue, the intense shear and localized heat produced during the UW process could potentially result in the degradation of phenolic rings or oxidation of hydroxyl groups in phenolic compounds [32]. This contrasts with the less mechanically abrasive, continuous direct contact heating process employed in HW, which facilitates enhanced retention and extraction of phenolic chemicals found in the plant tissue. The elevated TPC in the HW samples (98.33 mg GAE/g) compared to the UW samples (87.79 mg GAE/g) furnishes quantitative evidence that substantiates this hypothesis and elucidates the evident correlation between antioxidant activity across the ABTS, DPPH, FRAP and TPC levels. The higher level of antioxidant activity observed in HW samples may be attributed to the co-extraction of phenolic compounds under prolonged heat conditions, rather than being directly correlated with beta glucan content alone.

### 3.10. Inhibitory Activities of α-Amylase and α-Glucosidase

**Figure 6:**
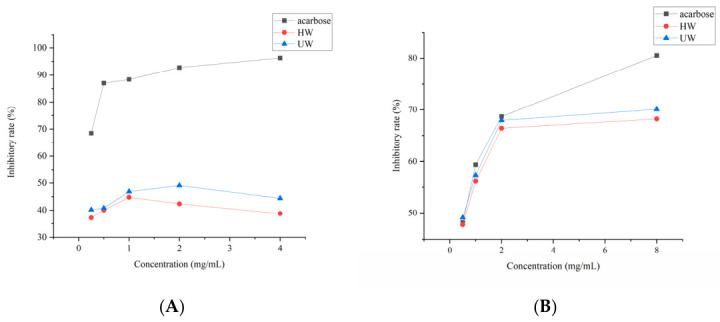
Enzyme inhibitory activities of crude polysaccharides. (**A**) α-Amylase inhibition. (**B**) α-Glucosidase inhibition.

The examination of the inhibitory efficacy of crude polysaccharide from A. sativa, extracted using HW and UW methods, against carbohydrate-related enzymes, specifically α-amylase and α-glucosidase, in comparison to the standard acarbose at different concentrations, demonstrated a notable inhibitory pattern with varying mechanistic significance, as illustrated in Figure 6.

In terms of α-amylase inhibitory activity, both HW and UW samples exhibited markedly lower percentage inhibition than acarbose across the examined concentration range (0.25–4 mg/mL). Acarbose demonstrated a peak inhibitory activity of 96.32%, while UW and HW samples reached maximum inhibitory activities of about 49.09% and 44.76%, respectively, constituting approximately 51% of acarbose’s inhibitory effectiveness. The inhibitory activity of both samples exhibited a non-dose-dependent plateau, reaching peak levels at moderate concentrations (1–2 mg/mL) before significantly diminishing at the highest concentrations. This may relate to polysaccharide aggregation at elevated concentrations, diminishing the surface area accessible for binding to the enzyme’s active site. The α-glucosidase inhibitory activity of the two samples exhibited a markedly distinct effect. The inhibitory efficacy of crude polysaccharide was comparable to that of acarbose over the examined concentration spectrum (0.5–8 mg/mL). Acarbose had 80.52% inhibitory activity at its maximum concentration, whereas the UW and HW samples displayed inhibitory activities of 70.00% and 68.00%, respectively, accounting for roughly 87% of acarbose’s inhibitory efficacy. This distinctly demonstrates a distinction in inhibitory activity levels that are nearer to the standard compound compared to α-amylase. The disparity in inhibitory activity between these two enzymes demonstrates the selective inhibition characteristic of crude polysaccharide; specifically, the extract displayed a significant inhibitory effect on α-glucosidase, like the standard compound, while exhibiting a diminished inhibitory effect on α-amylase [62]. The experimental findings align with previous research on fucoidan, a sulfated polysaccharide derived from seaweed. Fucoidan extracted from *Undaria pinnatifida* had markedly superior α-glucosidase inhibitory action compared to its inhibitory effects on α-amylase and Amyloglucosidase, making it a promising candidate for incorporation into bread to aid in reducing blood sugar levels [63]. Nevertheless, this could be evaluated alongside the monosaccharide composition analysis findings from the previous study, which indicated elevated glucose levels in both crude extraction methods. The glucose appears to originate primarily from the co-extraction of starch with beta glucan, rather than from beta glucan alone, presumably because starch serves as the natural substrate for both α-amylase and α-glucosidase. Consequently, the detected enzyme inhibitory activity in this study represents the aggregate of enzyme interactions with all constituents in the crude polysaccharide matrix, rather than the influence of pure beta glucan alone, when contrasting the two extraction procedures. The UW sample had superior inhibitory efficacy against both α-amylase and α-glucosidase in comparison to the HW sample. This aligns with the %Yield and beta glucan content comparisons detailed in the preceding section, which demonstrated that the UW approach produced a markedly higher beta glucan content in the extract compared to the HW method. This elevated beta glucan content in the UW sample is likely a key factor contributing to its marginally superior inhibitory effect against enzymes involved in carbohydrate breakdown, compared to the HW sample.

### 3.11. Practical Food Application Feasibility

The initial studies into the structural and functional characteristics of crude polysaccharide extracts from Thai-grown oats indicate their promise as an alternative raw material for the health food industry. The variations in physical properties and biological activity between two extraction methods may provide essential insights to select the best-suited method for specific product forms. Crude polysaccharide extracts from UW indicate favorable economic potential for efficiency and manufacturing costs, according to their higher extraction yield and beta-glucan content. This information may support assessments of future industrial scale-up to decrease unit raw material expenses [64]. In contrast, the crude extract from HW demonstrates notable food technology features, especially its improved swelling capacity. This characteristic is expected to serve as a texture modifier, viscosity enhancer, or water-retaining agent in food items requiring high stability, such as high-fiber baked goods, yogurt, or ready-to-eat soups [65,66]. Moreover, the enhanced TPC and antioxidant activity in the HW extract may help prevent rancidity caused by lipid oxidation, thus preserving quality and prolonging the shelf life of processed food products [8,67]. The α-glucosidase inhibition demonstrated by both extraction procedures may indicate the possible use of this extract in low-GI functional food products or alternative snacking [68,69]. The limited understanding of enzyme kinetics and the lack of comprehensive data on inhibition mechanisms constrain this research. This research is constrained by a limited understanding of enzyme kinetics and a lack of comprehensive data on inhibition mechanisms. The findings are only derived from in vitro testing and are likely related to the cumulative biological effects of the crude matrix, resulting from the synergistic interactions among the beta-glucan network, phenolic chemicals, proteins, and starch extracted sequentially. The specific effect of beta glucan alone cannot be identified. The explanation of the mechanism in this section relies on trends documented in the academic literature for closely related plant groups, necessitating further investigation and purification to validate the hypothesis.

## 4. Conclusions

This investigation evaluated the efficacy of crude polysaccharide extraction from *A. sativa* utilizing the HW and UW methods. The findings indicated that the UW methods produced a much higher %yield and beta glucan content compared to the HW method. This phenomenon is likely ascribed to acoustic cavitation, which appears to compromises the integrity of the cell wall structure, as suggested by structural findings from FTIR and SEM analyses. Monosaccharide analysis showed the existence of starch contamination in the crude extract, alongside naturally occurring beta glucan throughout the impurity extraction procedure. Nevertheless, the HW method produced superior outcomes for swelling power and antioxidant activity, which could be attributable to the disruption of polysaccharide chains and phenolic compounds caused by the strong shear forces in the UW method. Furthermore, extracts from both procedures demonstrated substantial α-glucosidase inhibitory activity, similar to acarbose, although they exhibited markedly reduced α-amylase inhibitory activity. This characteristic may be advantageous for regulating postprandial blood glucose levels while reducing gastrointestinal adverse effects. In conclusion, the extraction process selection must consider the target application. For optimal beta glucan yield and quantity, UW appears to be a highly suitable choice. However, considering functional characteristics like water-holding capacity and antioxidant activity, HW retains advantages that merit consideration. Future studies should examine its molecular weight to validate the chain-breaking mechanism and investigate the elimination of starch impurities to produce higher purity beta glucan products.

## Figures and Tables

**Table 1:** Hot water extraction method factors used in the BBD matrix for beta glucan content.

Run	A: Solid–Liquid Ratio (g/mL)	B: Extraction Temperature (°C)	C: Extraction Time (h)
1	1:20	50	30
2	1:40	50	30
3	1:20	90	30
4	1:40	90	30
5	1:20	70	15
6	1:40	70	15
7	1:20	70	45
8	1:40	70	45
9	1:30	50	15
10	1:30	90	15
11	1:30	50	45
12	1:30	90	45
13	1:30	70	30
14	1:30	70	30
15	1:30	70	30

**Table 2:** Ultrasound-assisted water extraction method factors used in the BBD matrix for beta glucan content.

Run	A: Solid–Liquid Ratio (g/mL)	B: Amplitude (%)	C: Extraction Time (h)
1	1:20	50	30
2	1:40	50	30
3	1:20	70	30
4	1:40	70	30
5	1:20	60	15
6	1:40	60	15
7	1:20	60	45
8	1:40	60	45
9	1:30	50	15
10	1:30	70	15
11	1:30	50	45
12	1:30	70	45
13	1:30	60	30
14	1:30	60	30
15	1:30	60	30

**Table 3:** Extraction yield and beta glucan content (BG) from *A. sativa* by Hot water extraction (HW) and Ultrasound assisted water (UW).

Run	Hot Water Extraction (HW)		Ultrasound Assisted Water (UW)	
	%Yield-HW	BG-HW	%Yield-UW	BG-UW
		(g/100 g)		(g/100 g)
1	33.05 ± 3.25	1.04 ± 0.17	61.87 ± 3.77	1.58 ± 0.49
2	35.90 ± 4.10	0.99 ± 0.06	67.09 ± 1.77	1.51 ± 0.24
3	43.83 ± 3.37	1.27 ± 0.21	71.56 ± 2.45	1.75 ± 0.27
4	49.03 ± 2.00	1.20 ± 0.20	75.51 ± 4.04	1.72 ± 0.61
5	23.85 ± 1.42	1.02 ± 0.15	73.70 ± 2.45	1.39 ± 0.33
6	24.09 ± 5.57	0.84 ± 0.16	71.46 ± 6.11	1.38 ± 0.12
7	37.82 ± 4.25	1.05 ± 0.73	83.66 ± 3.05	1.73 ± 0.24
8	43.79 ± 5.07	0.85 ± 0.09	74.38 ± 3.55	1.71 ± 0.35
9	25.74 ± 6.37	0.88 ± 0.14	59.72 ± 4.86	1.32 ± 0.21
10	39.37 ± 2.53	1.06 ± 0.33	70.96 ± 1.98	1.44 ± 0.37
11	35.30 ± 2.71	1.08 ± 0.15	69.52 ± 5.88	1.60 ± 0.36
12	43.40 ± 2.43	1.09 ± 0.12	79.38 ± 1.26	1.73 ± 0.35
13	37.08 ± 2.36	0.90 ± 0.17	74.53 ± 2.67	1.60 ± 0.07
14	38.95 ± 4.68	0.88 ± 0.22	72.26 ± 5.19	1.69 ± 0.63
15	39.87 ± 1.49	0.84 ± 0.15	73.50 ± 5.74	1.66 ± 0.29

**Table 4:** Regression analysis of %yield and beta glucan content from hot water extraction (HW).

Source	Yield-HW (g/100 g)		BG-HW (g/100 g)	
	Sum of Squares	Prob > F	Sum of Squares	Prob > F
Model	726.29	0.0208	0.23	0.0393
A	25.41	0.1906	0.03	0.0484
B	260.41	0.0047	0.05	0.0228
C	279.15	0.0040	0.01	0.2013
A^2^	2.81	0.6359	0.02	0.0714
B^2^	26.76	0.1811	0.11	0.0049
C^2^	106.54	0.0269	0.00	0.6783
AB	1.39	0.7380	0.00	0.9256
AC	8.20	0.4291	0.00	0.8565
BC	7.63	0.4448	0.01	0.2874
R^2^	0.9290		0.9064	
Lack of fit	0.1072		0.1497	
Adj R^2^	0.8013		0.7379	
C.V.	9.07		6.86	

A = ratio; B = temperature; C = extraction time.

**Table 5:** Regression analysis of %yield and beta glucan content from ultrasound-assisted water extraction (UW).

Source	Yield-UW (g/100 g)		BG-UW (g/100 g)	
	Sum of Squares	Prob > F	Sum of Squares	Prob > F
Model	442.51	0.0467	0.29	0.0021
A	3.59	0.5741	0.00	0.3006
B	163.91	0.0097	0.05	0.0027
C	120.99	0.0175	0.19	0.0001
A^2^	0.50	0.8321	0.00	0.5767
B^2^	113.27	0.0198	0.00	0.4513
C^2^	14.79	0.2772	0.04	0.0034
AB	4.56	0.5284	0.00	0.6138
AC	12.39	0.3153	0.00	0.8325
BC	0.47	0.8365	0.00	0.9077
R^2^	0.8989		0.9731	
Lack of fit	0.0768		0.6532	
Adj R^2^	0.7170		0.9247	
C.V.	4.4		2.53	

A = ratio; B = %amplitude; C = extraction time.

**Table 6:** Validation of optimized extraction conditions for beta glucan yields across different methods.

Conditions	Optimized Process Parameters	Predicted %Yield	Actual %Yield	%Error	Predicted β-Glucan (g/100 g Dry Extract)	Actual β-Glucan (g/100 g Dry Extract)	%Error
HW	1:20, 90 °C, 35 min	44.23	41.32 ± 0.98	6.58	1.27	1.25 ± 0.27	1.57
UW	1:20, 65% amplitude, 45 min	83.36	82.72 ± 2.19	0.77	1.76	1.75 ± 0.87	0.57

**Table 7:** Antioxidant and total phenolic effectiveness of each condition.

Conditions	ABTS (µmol TE/g Sample)	DPPH (µmol TE/g Sample)	FRAP (µmol TE/g Sample)	TPC (mg GAE/g Sample)
HW	0.67 ± 0.04	0.53 ± 0.06	0.05 ± 0.02	98.33 ± 7.68
UW	0.58 ± 0.06	0.35 ± 0.06	0.03 ± 0.01	87.79 ± 3.07

## Data Availability

The original contributions presented in the study are included in the article, further inquiries can be directed at the corresponding author.

## Abbreviations

The following abbreviations are used in this manuscript:

HW	Hot water extraction
UW	Ultrasound-assisted water
BG	Beta glucan
